# More fishers and fewer martens due to cumulative effects of forest management and climate change as evidenced from local knowledge

**DOI:** 10.1186/s13002-017-0180-9

**Published:** 2017-09-07

**Authors:** Pauline Suffice, Hugo Asselin, Louis Imbeau, Marianne Cheveau, Pierre Drapeau

**Affiliations:** 1 Université du Québec en Abitibi-Témiscamingue, 445, boulevard de l’Université, Rouyn-Noranda, Québec, J9X 5E4 Canada; 2 Ministère des Forêts, de la Faune et des Parcs, Gouvernement du Québec, 880, chemin Sainte-Foy, 2e étage, Québec, Québec G1S 4X4 Canada; 3 Université du Québec à Montréal, 405 Rue Sainte-Catherine Est, Montréal, Québec, H2L 2C4 Canada

**Keywords:** Mustelids, Trappers, Aboriginal people, Anthropogenic disturbances, Snow conditions

## Abstract

**Background:**

Monitoring of fur-bearing species populations is relatively rare due to their low densities. In addition to catch data, trappers’ experience provides information on the ecology and status of the harvested species. Fisher (*Pekania pennanti*) and American marten (*Martes americana*) are mustelids that are sensitive to forest management and therefore considered to be ecological indicators of forest health. Fisher populations have increased in eastern North America since the early 2000s and this could have resulted in a northeastern extension of the species’ range and increased overlap with marten’s range. Moreover, habitats of both species are subject to natural and anthropogenic disturbances. The objective of this study was to document the knowledge held by local trappers in the northern area of sympatry between fisher and marten to identify factors that could explain variation in populations of the two species and interactions between them.

**Method:**

Forty-one semi-directed interviews with Indigenous and non-Indigenous trappers in the Abitibi-Témiscamingue region of western Quebec (Canada), at the northern limit of the overlapping ranges of the two mustelid species.

**Results:**

Trappers highlighted the lack of exclusivity of marten and fisher to coniferous forests, although marten is more closely associated with them than is fisher. Fisher apparently also takes advantage of open environments, including agroforestry systems. Moreover, climate change increases the frequency of freeze-thaw events that cause the formation of an ice crust on the snow surface, which favors fisher movements.

**Conclusion:**

The fisher was identified as a competitor and even a predator of the marten. Furthermore, the fisher is less affected than the marten by forest management, and it also seems to benefit from climate change to a greater extent.

## Background

Fur-bearing mammals are considered to be particularly sensitive to habitat loss and fragmentation [1–4]. Their habitats have been affected especially by major changes that are incurred through human activities, such as forest harvesting and agricultural development [5, 6]. Climate change could further modify or reduce the quality of available habitats [7, 8]. Yet climate change and anthropogenic disturbances such as forest harvesting may also increase access to new territories by modifying biotic and abiotic factors that otherwise would limit a species’ potential to utilize a territory [9]. Given that climate change and anthropogenic disturbances occur over long periods of time, resulting in complex cumulative impacts, they are often difficult to understand and to document [10, 11].

Populations of fur-bearing animals typically exhibit low densities. Consequently, monitoring these species to document their status is relatively rare [12, 13]. Sales of trapped pelts have long been used by wildlife managers to track fluctuations in the abundance of certain wildlife populations [14–16]. However, from one year to the next, fur sales can be influenced by animal population status and trapping efforts, which depend upon numerous social (e.g., employment-trapping conciliation, trappers’ health status), economic (e.g., variation in fur prices, available material resources), and environmental (e.g., weather, local habitat disturbance) factors [17–19]. Beyond information that is provided by fur sales, the experience of trappers and the knowledge that they accumulate over many trapping seasons is an invaluable, frequently untapped source of information, which would enrich our understanding of species with relatively low densities, such as mustelids [20–22].

The growing interest in local knowledge is due, among other things, to the potential saving on time and money needed to gather the scientific information required to meet environmental challenges [23, 24]. In addition to the number of individuals that are caught, trappers’ experience allows us to learn more about the ecology of exploited species [22, 25]. Trappers continuously monitor population dynamics as well as natural and anthropogenic forest disturbances. Trappers have developed a solid expertise regarding ecosystem responses, including wildlife responses to habitat changes, over broad spatiotemporal scales [26, 27]. Knowledge on habitat requirements of those species which are most sensitive to forest practices, from both local and scientific sources, is essential for the conservation of key habitats [28].

Both fisher (*Pekania pennanti*) and American marten (*Martes americana*) are mustelids trapped for their fur that play a key role in forest social-ecological systems in eastern North America. They are sensitive to forest management prescriptions and considered to be ecological indicators of forest health [1, 29, 30]. American marten is one of the species most frequently sought by trappers because of its ease of capture and the high market value that is placed upon its fur [20, 31, 32]. In addition, marten and fisher are important in some Indigenous cultures, notably those of the Anishnaabeg [33] and the Cree [34]. These species can be considered as cultural keystone species that are essential in maintaining the complexity of socio-ecological systems [35].

During the 1970s, the fisher experienced periods of low abundance in North America [36]. This was attributed to over-exploitation of its fur and habitat loss [36]. Since the early 2000s, the populations appear to have recovered and the interest of trappers in the fisher has intensified, resulting in a substantial increase in fisher pelt sales, especially in Quebec [15]. Sales of fisher pelts apparently indicate that their geographical distribution could be expanding towards the northeast. This change in fisher distribution would thus result in greater overlap with marten’s range in habitats that are undergoing natural [37] or anthropogenic disturbances [6, 38]. The objective of this study was to document the local knowledge of trappers in western Quebec (Canada), where distributions of fisher and marten are sympatric, to identify factors that could explain variation in populations of the two species and the interactions between them.

## Methods

### Study area

Fisher and marten are two species that are endemic to North America. The geographic distribution of the fisher is the least extensive of the two species, and straddles the southern portion of marten’s range [39]. Fisher is found mainly in temperate and boreal forests of North America. Since the mid-1800s, the geographic distribution of the fisher has decreased considerably, primarily due to changes associated with the Little Ice Age (ca. 1250–1750 CE) in eastern North America [40].

**Fig. 1:**
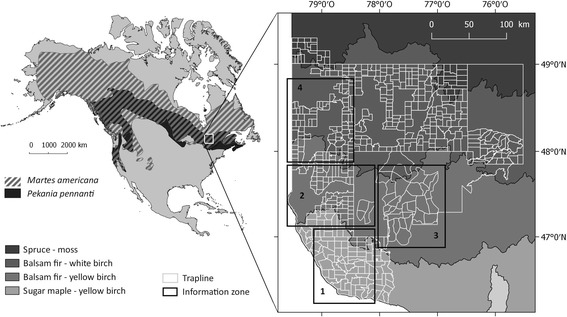
- Location of the study area in the northern part of the geographic range where fisher (solid) and American marten (hatched) overlap. The study area is structured into traplines that are distributed in the 3 bioclimatic domains. Also shown are the clustering areas of participants having provided similar information [Source of ranges: http://www.natureserve.org]

The study was located in Abitibi-Témiscamingue, western Quebec, at the northern limit of the overlapping ranges of the two mustelid species (Fig. 1). According to climate change scenarios, snowpack thickness should decrease and spring rainfall will likely increase in the region [41]. The study area covers a latitudinal gradient encompassing three bioclimatic domains: sugar maple-yellow birch; balsam fir-yellow birch; and balsam fir-white birch [42].

The sugar maple-yellow birch bioclimatic domain occupies the northernmost part of the temperate deciduous zone. The main overstory species are sugar maple (*Acer saccharum* Marsh.), yellow birch (*Betula alleghaniensis* Britton), red oak (*Quercus rubra* L.), eastern hemlock (*Tsuga canadensis* [L.] Carrière), white pine (*Pinus strobus* L.) and red pine (*Pinus resinosa* Aiton). Després et al. [43] have shown that frequent and small gaps are the main natural disturbance agents in this area. In this bioclimatic domain, fisher represents 40% of the combined sales of marten and fisher pelts (10-year average, 2006 to 2015) [44].

The balsam fir-yellow birch domain corresponds to the transition between the northern temperate zone and the boreal zone. The main tree species are yellow birch and conifers, such as balsam fir (*Abies balsamea* [L.] Mill.), white spruce (*Picea glauca* [Moench] Voss), pines (*Pinus* spp.), and eastern white cedar (*Thuja occidentalis* L.). Spruce budworm (*Choristoneura fumiferana*) outbreaks and catastrophic wildfires are the two main natural disturbances [45, 46]. In this sub-domain, fisher constitutes 17% of the combined sales of marten and fisher pelts.

The balsam fir-white birch domain occupies the southern part of the boreal zone. The forest landscape is dominated by fir and white spruce stands, with paper birch (*Betula papyrifera* Marshall), trembling aspen (*Populus tremuloides* Michx.), black spruce (*Picea mariana* [Mill.] Britton, Sterns & Poggenb), jack pine (*Pinus banksiana* Lamb.), eastern larch (*Larix laricina* [Du Roi] K. Koch), and white cedar. As is the case in the balsam fir-yellow birch domain, spruce budworm and fire are the main natural disturbances [47–49]. In this domain, marten dominates fur sales, with fisher accounting for about 8% of combined sales. In the three bioclimatic domains, logging has become a major source of disruption over the last few decades (e.g., [50]).

Despite a sharp downturn in fur prices over the past few decades, together with habitat loss and the development of fur farms, trapping activities still generate substantial revenues [18, 32]. The province of Quebec registers the highest sales of American marten (15 to 30%) and fisher (25 to 33%) pelts in Canada [51]. In Quebec, about 8000 trapping licenses have been purchased annually since 2010 [52]. Commercial trapping is practiced in three categories of territory: structured (traplines), unstructured (open access) and restricted access to Indigenous people only. Abitibi-Témiscamingue is one of the most popular regions for trapping in Quebec; in 2012, the economic benefits (gross domestic product, tax revenues) generated by trappers, weighted by population, were the highest in the province [32].

**Fig. 2:**
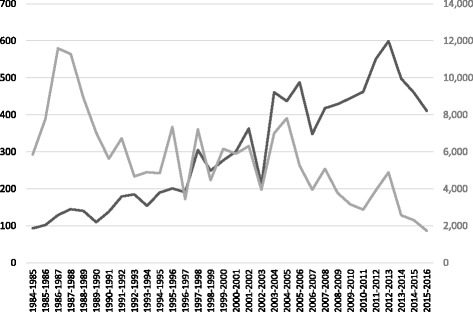
– Number of fisher (black) and American marten (grey) pelts that were sold per trapping season in Abitibi-Témiscamingue (Marianne Cheveau, MFFPQ, Unpublished Data)

The study area straddles the traditional territory of several Indigenous communities for which trapping is an important cultural and subsistence activity [53, 54]. Some trappers in the region are concerned about a decline in marten catches, which have been observed with concomitant increases in fisher catches (Fig. 2). Other trappers are concerned about a decrease in fishers in some parts of the region. Our preliminary conversations with trappers (both Indigenous and non-Indigenous) have suggested intra-regional variability in the evolution of fisher and marten abundances. Trapline area averages 100 km^2^ in Abitibi-Témiscamingue (minimum area = 21 km^2^ and maximum area = 643 km^2^ for 498 traplines).

### Recruitment of participants

Trapping is an activity that requires knowledge of the biology and ecology of furbearers. This knowledge develops with experience over the years. The use of land and natural resources thus fuels trappers’ knowledge [55]. To ensure the sustainability of their harvests, trappers must pay particular attention to the sampling effort that they deploy under changing environmental conditions [56]. Traplines are thus considered to be integral ecosystems that must be managed responsibly [54]. Trappers are recognized as “land custodians,” who are particularly interested in documenting changes in the distribution and harvesting of fur-bearing animals and the effects of habitat disturbances on forest operations and climate change [56]. We sought to meet with experts, both Indigenous and non-Indigenous, who were the most recognized by their peers because of their experience in trapping and their knowledge of the forest in general, and the habitats of marten and fisher in particular. We have created four groups: i) marten trappers who were active on the same territory for several years (active trappers, AT); ii) trappers who were recognized as experts by their peers, but who are no longer able to carry out their activities on a full-time basis because of their age (recommended trappers, RT); iii) experts who were involved in the management of trapping areas or in fur sale (experts, Ex); iv) elders (Elders, E). Inclusion of elders has been justified, given their important role in the transmission of forest-related knowledge over the generations, and which has increased the temporal depth of information. We used the “snowball sampling method,” whereby people in the communities identified the most experienced trappers, who in turn were able to suggest other trappers, and so on [57, 58]. This identification of local experts by peers ensures the recruitment of the best-informed individuals [57, 59].

### Data collection and analysis

We interviewed the participants in semi-directed individual interviews [60]. This is a flexible survey technique that offers possibilities for acquiring additional information, within a familiar exchange framework [61]. We conducted 41 interviews with Indigenous (*n* = 27) and non-Indigenous (*n* = 14) trappers. Indigenous trappers were members of the Anishnaabeg communities of Kebaowek (*n* = 5), Kitcisakik (*n* = 17), Timiskaming First Nation (*n* = 4) and Wolf Lake (*n* = 1). Most participants were men (*n* = 38) over the age of 40 (*n* = 37). Geographic areas were delineated a posteriori in order to group participants with consistent information: Zone 1 (*n* = 8), Zone 2 (*n* = 14), Zone 3 (*n* = 14) and Zone 4 (*n* = 4) (Fig. 1). The activities of one participant covered the entire study area. Interviews were conducted in French, English or Anishnaabemowin, depending upon the preference of each participant. Anishnaabemowin interviews were conducted in the presence of a contact person with whom the participants were familiar and who could provide translation. Codes that were used to identify participants corresponded to their status (AT, RT, Ex or E), followed by a number corresponding to the order in which they were encountered, and the area to which they could relate (Z1, Z2, Z3, Z4).

The interviews were intended to provide a better understanding of trappers’ perceptions of marten and fisher habitat use, and the interactions between the two species. The purpose of the questions that were asked was to document the perspectives of trappers on marten and fisher ecology within the study area and their perceptions regarding the effects imposed by forest management and changes in snow cover (Appendix). The main themes of the interviews were: (1) predator-prey relationships of marten and fisher; (2) the evolution of annual harvest success; (3) characteristics of locations that were utilized by each species, including hunting, cover and breeding sites; (4) the effects of forest management on habitats; and (5) changes in winter conditions. Interviews were conducted between August 2015 and March 2016. Participants were re-contacted following the analysis of the individual interviews to complete and validate the information [62]. Thematic analysis of the interviews was conducted using QSR NVivo 10 software, which made it possible to divide the corpus of information into themes to define its meaning by successive destructuring-restructuring operations [63]. Precise identification of species that were mentioned by the participants was not always possible. They are presented at the most precise taxonomic level possible, i.e., according to genus or family.

### Ethical considerations

This project has responded to a request from Indigenous communities to investigate and better understand the reasons behind fluctuations in fisher and marten populations in their territories. We received a certificate from the Ethics Review Board at UQAT (the Université du Québec en Abitibi-Témiscamingue, # 2015–04). The project was also approved by the Band Councils of the participating communities: Kitcisakik, Timiskaming First Nation, Kebaowek and Wolf Lake. Each meeting began with a presentation of the context of the study and the rules of confidentiality. Participants were asked for their consent prior to the interview. Participants were involved in this research on a voluntary basis. Participants who wished received a summary of the main results of the research. Preliminary results were presented to the participating communities in advance of manuscript preparation and submission.

## Results

### Predator-prey relationships

#### Marten prey species

**Fig. 3:**
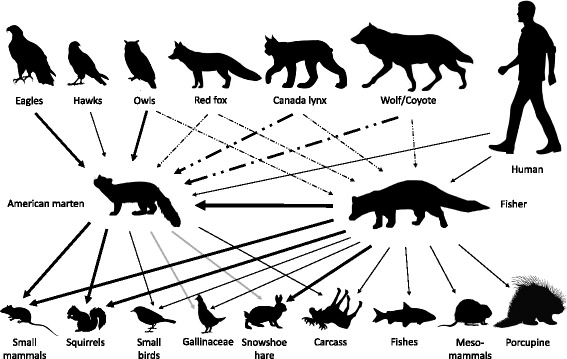
- Predator-prey relationships of American marten and fisher, based on local knowledge. Line thickness indicates the relative importance of the relationship. Grey lines indicate a winter relationship, while dotted lines indicate predation exclusively on young animals. [Source of images: www.shutterstock.com]

According to the participants, the marten feeds primarily on squirrels and other small mammals (Fig. 3). The term “mouse” was used generically to refer to several small mammals, including voles, shrews and other unspecified species. Squirrels included several species of Sciuridae, such as the red squirrel (*Tamiasciurus hudsonicus*), the large flying squirrel (*Glaucomys sabrinus*) and the striped chipmunk (*Tamias striatus*). Marten also hunt snowshoe hare (*Lepus americanus*) and gallinaceous birds, including ruffed grouse (*Bonasa umbellus*) and spruce grouse (*Falcipennis canadensis*), especially in winter. Ruffed grouse catches were attributed to hunting under the snow, but the marten also consumes the eggs and preys upon the young of this species. Further, participants felt that the marten’s ability to climb trees also allowed it to feed upon small perching birds. Other dietary components that were cited by participants included carcasses of moose (*Alces americanus*) and beaver (*Castor canadensis*), animals that had been caught in traps, and red raspberries (*Rubus idaeus* L.).

[The marten, she eats a lot of mice. A year where you have a lot of mice – and this is a 3-year mouse cycle – the marten will raise four young, (…) but the following year you will have practically no babies. The squirrel is also a feast for the marten: when you have the marten, you do not hear squirrel calls.] [E1-Z2]

[People think that the marten catches hares, partridge, but it is mostly the mouse that it hunts. When there is too much snow, she switches to the air (in the trees) to catch squirrels, flying squirrels.] [AT12-Z2]

#### Fisher prey species

Participants have pointed out that the fisher is very opportunistic and eats whatever it encounters. It is a known predator of the North American porcupine (*Erethizon dorsatum*), but this species is not its main food source. Like the marten, the fisher hunts hare, squirrels, gallinaceous birds, small mammals, and small birds. Because of its size and strength, the fisher can access a greater diversity of prey than can marten, including (semi-)aquatic animals (fish, beaver, muskrat [*Ondatra zibethicus*], mink [*Neovison vison*]) and larger-sized terrestrial animals, such as woodchucks (*Marmota monax*), striped skunk (*Mephitis mephitis*), farm chickens (*Gallus gallus domesticus*), domestic cats (*Felis silvestris catus*), and even red fox (*Vulpes vulpes*). Fishers also feed upon moose carcasses or animals caught in traps, including martens. Most participants also pointed out that the fisher also eats live martens and is one of the latter’s principal predators. Like the marten, the fisher also eats berries.

[(The fisher) even eats fish, it goes up to whitefish spawning grounds, where it often drags fish ashore, like the lynx. The marten she does not do that, she is not able to kill a fish in the water.] [Ex30-Z3]

[(The fisher) is the only animal that is going to eat porcupine, it is able to go and kill it, it flips it on its back and seizes the prey by the neck.] [AT2-Z1]

#### Relationship between the marten and fisher

Fisher and the marten share the same “pantry” and compete for the same resources, but the fisher also exerts direct predation pressure upon the marten. In addition to martens that have been caught in traps and eaten by a fisher, as evidenced by tracks around the carcasses, the trappers supported their observations based upon the tracks of fishers that appeared to chase down the martens. In Indigenous communities, this is common knowledge that has been passed down from generation to generation, since the participants mentioned that "this is what the elders say." Several trappers have recalled that the fisher, through its strength, agility and rapidity, has the ability to chase and catch marten. Participants have reported that the more fishers that are observed on a territory, the fewer the number of martens.

[Clearly, it is the fisher that has the upper hand. It is faster on the ground, but the marten is faster when climbing trees. But the marten, sometimes, it jumps from tree to tree and misses its mark. The fisher waits on the ground beneath the tree. If the marten falls to the ground, it is done for, it is so fast! In the snow, the marten is going to walk under windthrow, but the fisher struggles more, it is too big. That is why the marten loves the windthrow, the overturned wood, the old roots. The fisher will go there too, but it lacks the facility of the marten.] [AT8-Z1].

[But fishers also eat martens. They kill martens. I saw that they chase them, from the tracks, the fishers run after the martens.] [RT38-Z1]

#### Predators

Most participants identified the fisher as the principal predator of the marten. Other predators of marten that were recognized by participants were, in decreasing order of frequency: raptors, grey wolf (*Canis lupus*), Canada lynx (*Lynx canadensis*), red fox, coyote (*Canis latrans*), the domestic dog (*Canis lupus familiaris*) and other martens. Among the birds of prey, some participants noted that they were mostly owls, including great horned owl (*Bubo virginianus*), together with eagles (bald eagle, *Haliaeetus leucocephalus*; golden eagle, *Aquila chrysaetos*), falcons and hawks. Participants pointed to increased observations of bald eagles, golden eagles and owls. For wolves, coyotes, foxes and lynxes, participants indicated that these would mainly prey on young or martens caught in traps, while the fisher would probably have difficulty catching live adults. The presence of coyote is relatively recent in the region, since it arrived in Témiscamingue (zones 1 and 2) about 15 years ago. Trappers have indicated that it is mainly concentrated in agroforestry environments. They also pointed out that wolf and lynx populations have been increasing throughout the region for several years. Participants had more difficulty identifying predators of the fisher. They consider that because of its size, rapidity, ferocity and ability to climb trees, it can flee most other carnivores. However, they pointed out that fisher mortality must occur mainly among the young, which can be caught more easily by canids such as wolves, coyotes and foxes, or by lynxes. Some have mentioned that wolves and coyotes mostly eat fishers when these are caught in traps. Other potential sources of mortality in fishers that were cited by participants are human, birds of prey, mainly great horned owl that preyed upon the young, and infections that were incurred from porcupine spines. It has also been mentioned that fishers eat one another when defending their territories where they are very numerous. Several trappers reported that fisher and marten populations were also influenced by competition from lynx.

[The little ones (fishers) surely have predators, but not the adults.] [RT29-Z3]

[The wolf and coyote are able to attack (the fisher) quite easily because the wolf attacks pretty much everything that moves. When you have a lot of wolves on a territory, there is hardly anything else.] [AT4-Z2]

### Evolution of captures

#### Marten captures

Participants have all found that marten captures have been declining for several years, but more markedly in the 2015–2016 season. The trappers were unanimous in stating that they have observed far fewer tracks recently compared to previous seasons. These responses point to several underlying causes. First, participants mentioned fluctuations in trapping effort, which intensify when the price of pelts from the previous season was high. Second, some trappers admitted to having captured a large number of martens in the 1980s, and observed mainly females in subsequent years, which can be interpreted as an indicator of over-exploitation. Third, several participants stressed the ease with which marten could be caught in traps, together with the increased likelihood of over-trapping them quickly when capture regulation was not yet implemented. Fourth, when prices for marten pelts were high, participants had noted an increase in competition by more occasional trappers. Since then, prices have dropped substantially and trapping has decreased. Several trappers suggested that fur price on the marketplace was the main factor regulating marten catches.

Several trappers have suspected severe winters (e.g., 2014 and 2015) as being potentially responsible for the decline in marten populations. Participants further attributed the general decline in marten populations to changes in their habitats and to direct mortality incurred by forest harvesting machinery. Participants underscored the fact that after logging, game can desert the territory due to the lack of protection, thereby reducing the prey base upon which marten relies. Further, they explained that marten avoids crossing large canopy openings, such as those created by harvesting operations; rather, it prefers to move along the edges of intact forest.

According to participants, the number of captured martens fluctuates with food availability, especially the abundance of small mammals. In years when trappers observed many small mammals, they found that the marten was not sufficiently hungry to explore the territory and potentially fall prey to a trap. Indeed, martens moved less since they had access to abundant food which could reduce the attractiveness of bait presented in a trapping device [16]. In contrast, participants were surprised that in recent years, even after a year of high small mammal abundances, marten captures no longer increased. They often referred to the asynchronous cycles of lynx and snowshoe hare as a model for marten and small mammals. Some stated that there would be a 2-year lag between a small mammal peak and a marten peak. A link was also made with ruffed grouse, but the suggested agreement between cycles here was much less pronounced. In Zone 3, a decline in populations of both hares and ruffed grouse is the main explanation that accounted for the scarcity of martens, which can no longer find food in cutover territories. According to several participants, decline in marten catches apparently coincided with increases in fisher abundance, particularly in the northern part of the study area (Zones 2 and 4).

[A year where martens have no trouble finding food, it is going to be a year where people are going to catch less. When it has food, it will stay there, it will move less, is less in search. So it is going to have less opportunity to go through a trap. (…) A year like this year when the sampling was less good, I call it a year for nature. I'm happy with that.] [Ex9]

#### Fisher captures

Participants in Zones 2 and 3 (see Fig. 1) indicated that fisher populations had decreased, particularly in the late 1980s as a result of increased trapping efforts that were driven by high fur prices. At that time, they indicated that the price of fisher pelts was higher than that of marten. In addition, Zone 3 participants attributed reduction in fisher abundance to the cutting of red and white pine that this species utilized as resting sites or used for raising young. Participants in Zones 2 and 3 found that as a result of the aforementioned period, fewer fishers travelled during the 1990s. Since the mid-1990s, participants reported that numbers of captured fishers remained relatively stable in Zone 1 (Fig. 1), while they have been increasing since 2000 in Zone 2, increasing since 2010 in Zone 3, and increasing since 2007 in Zone 4. According to Zone 4 participants, fisher captures commenced in 2000. They attributed this development to fisher recovery in places that are difficult to access with forestry machinery, viz., on steep slopes and near rivers. In contrast, participants from Zone 2 noted that increases in fisher captures coincided with the arrival of raccoon (*Procyon lotor*) to the area. Raccoons are found predominantly in Zones 1 and 2, and more recently, in Zone 3. Zone 2 trappers have also noted that raccoon populations have increased, especially since the development of cereal crops (especially corn) in the zone.

Peaks in populations that had been observed by participants likely included incidental captures that were made in traplines originally planned for marten or fox capture. Peaks were also the result of changes in the type, size and layout of traps that were designed specifically to capture fishers. For example, trappers employ larger traps, which are situated closer to the ground. They recognized the risk of the trapped animal being damaged by small mammals or by overheating when their bodies came in contact with the soil, but stressed greater capture efficiency. Other trappers have even installed snares that are specifically made to trap fishers for only a few years, similar to those they would deploy for foxes. Some participants mentioned that indicators of fisher presence, such as captures or snow tracks, had decreased throughout the study area over the past 5 years; in some areas of Zones 1 and 3, these declines occurred over the past 10 years. One explanation mentioned by the participants was based upon reductions in fisher populations due to a substantial harvest by trappers. Other explanations were related to general changes in habitat and the possibility that severe winters could affect the survival and, therefore, the state of fisher populations.

### Habitat

#### Marten habitat

Most participants identified their best marten capture sites as being primarily located in conifer stands, especially spruce. Participants trapped martens in other conifer stands, which were dominated by fir, white cedar, pine, and hemlock, yet mixed forests were also suitable for marten capture. Trappers set up traps primarily in mature stands, viz., old forests where there are more branches, which they characterized as “dirty and dense forests.” While participants considered that martens used hardwood forests, these were more likely to be transient habitats. Indeed, trappers indicated that martens move with great difficulty through hardwood stands because of snow accumulation and, especially in winter, hardwood forests are too open to avoid predator attacks. According to the participants, good marten habitat is at the interface between coniferous and hardwood forests. Martens require cover to protect themselves from predators, but they also need to find food. Participants stressed the need for ground structures where small mammals can hide. Trappers further indicated that the presence of gaps or windthrows favors the presence of marten. This type of habitat structuring is mostly found in mature forests and on hillsides. Participants also explained that riparian areas were strategic locations for setting traps because martens often travel through these zones. Finally, participants emphasized the presence of old trees in which martens can make their dens, such as in woodpecker cavities or downed wood on the ground, and in old birch trees or snags.

#### Fisher habitat

Some participants attributed the same habitats to the fisher as to the marten, but several others emphasized the importance of large pine and white cedar utilization by the former species, particularly large hollow trees where fishers could take refuge. Participants also mentioned that fishers could travel greater distances than martens. Trappers likewise frequently set their traps along watercourses, which was part of the same strategy that was employed for capturing martens. Yet some trappers noted that more fishers could be caught in the hills, in contrast to results obtained for marten. Similarly, trappers found that they could catch more fishers than martens in deciduous forests. Unlike martens, fishers utilized cut-overs; indeed, fisher populations increased with the concomitant removal of forest canopies during logging. Trappers in Zone 2 reported successful fisher captures in a variety of habitats, ranging from mature forest to agroforestry landscapes, to edges of agricultural fields, and even in close proximity to human habitation rather than deep within forest stands. In the same area, trappers snared fishers in traplines that were intended for capturing foxes. Several trappers have indicated that fishers can use more sparse habitats than martens because they have less need for protective cover against predators. Experts have reported that trappers associate the presence of fishers with that of Canada lynx. They explained that the two predators share the same food resources, i.e., snowshoe hare and ruffed grouse.

### Forest management

#### Changes in forest structure and composition

Most trappers have deplored the adverse effects imposed by industrial forestry practices on the vegetation, which destroys the forest understory through the passage of harvesting machinery. Even when logging is followed by replanting, “game forests” are transformed into “deserted gardens.” Participants reported a marked change in forest composition in Zone 1 resulting from the exploitation of large coniferous species, such as white and red pines. They also noted a general increase in the proportion of hardwood tree species throughout the territory. Zone 4 participants have pointed out, however, that hardwood species do not provide good habitat for marten. All participants disapproved of the few old forests that were left intact by forest management. They explain that by changing the composition of forest species, the entire ecosystem has become impoverished.

[There is nothing in a plantation: no birds, no hares, it is a ghost forest.] [Ex16-Z2]

#### Effects on marten and fisher

Effects of forest management on marten and fisher that were cited by participants were related mainly to protective cover and food availability. Some trappers also mentioned that sun exposure of the animal coats makes the fur look pale. The fur of martens and fishers appears lighter in areas where the forest is more open, such as in hardwood stands and in cut-overs. In general, marten habitat would progressively become less adequate, but fishers appear to be capable of living in sparser forest stands. The return of marten following logging would appear, however, to be more rapid in mixedwood than in softwood stands. In the mixedwoods, regeneration would be faster, permitting marten return in 6 to 10 years. Trappers estimated that softwood stand would have to achieve a height of 12 ft (3.66 m) before marten return was noticeable. Yet habitat changes that are induced by forest harvesting would benefit certain species, such as snowshoe hare, moose and beaver, which settle more quickly after harvesting and proliferate in the region. One participant noted that for the last 10 years, he no longer perceived cyclic dips in hare populations. Forest management would also benefit lynx and wolves, the populations of which have been increasing throughout the region for several years. Participants remarked that increases in Canada lynx and grey wolf populations were exacerbated by limited trapping of both species for several years (bag limit on lynx, and a lack of trapper interest in the wolf).

[In cut forests, animals, if they have no shelter, they do not stay there.] [RT18-Z3]

[Because when it has been cut, the marten, you can forget about it. And, I’m going to tell you that right now, it is pitiful. It is the opposite for the fisher.] [AT11-Z2]

#### Orientation for development

Participants advocated that forest cover should be maintained not only to retain predator food sources, but also to protect the marten from "all predators that can run after it." They emphasized the importance of retaining pines and white cedars, in particular, as resting sites for marten and fisher. More generally, they stressed the need to conserve dead wood on site. They considered that protection strips (forest that is left untouched) around lakes and wetlands were not wide enough to act as appropriate buffers. Partial cuts were much more appreciated than clear-cuts by the trappers. Although they recognized that the forest industry currently maintains harvest retention patches, trappers did not consider that these were large enough to support game. Rather than leaving a plot of standing timber in the midst of a large clear-cut, participants suggested that smaller cuts surrounded by large-diameter trees promoting natural regeneration were preferable. They recommended a forest harvesting system, such as gaps or strip-cuts, which permit harvesting of forest patches, while retaining the forest matrix.

### Winter conditions

Participants stressed that they were attentive to climatic conditions, particularly weather that influence fur quality of the species that they harvest. They further indicated that snow conditions greatly influence the movements of all animals, as well as those of trappers. Winter characteristics determine the duration and intensity of trapping activity. Participants found that the seasons have shifted through time, with "the fall that is later and the spring that never wants to happen." In addition, most participants reported that over the past decade, the quantity of snow falling annually tended to decrease. However, some were more circumspect in their opinions, mentioning that it was difficult to ascertain whether there was a general trend, given large within- and between-year variation. All noted that winters were generally milder, however. Within the same winter, the amplitude of temperature variation becomes more and more important, more periods of thawing, sometimes accompanied by winter rains followed by intense cold. Lakes do not have time to freeze thoroughly, such that the surface ice layer is thinner than before. Trappers also mentioned that freeze-thaw events were much more frequent during the same winters. Large temperature variations favor the formation of an ice crust on the surface of the snow, thereby increasing the load-bearing properties of the snow for small animals. According to participants the period during which the crust was present has begun earlier and has become increasingly longer through winters. Soft snow is an important factor limiting the movements of the fisher, which then mainly uses “trampled corridors” left by snowshoe hares. In the presence of a crust, it is certainly easier for fishers to move about and hunt. However, several trappers noted that crusts have not been as thick as observed in the past. Participants also reported an increase in the frequency and intensity of winds, which caused more frequent large windthrows in recent years.

[There was not as much crust as that, before, the crust was just in springtime. Now it happens in winter, in December it has already happened. Before it was only March-April, when the sun starts to rise much higher above the horizon, it melts on top. Sometimes, it is too thin for us to walk on, but it does not bother the animals.] [AT22-Z2]

[I have always benefited from the crust, but it happens earlier now and it will last longer because we have had a lot of rain and it has permeated the snow from one side to the other. For a fisher, it must be paradise.] [AT4-Z2]

[If there is soft snow, the marten is going to have the advantage over the fisher that runs after him. On the hard (snow), I think the fisher would have the advantage.] [AT6-Z4]

[The best fur is when it is cold. Pelts are darker when taken later, and the same is true for all species. The price is better for dark fur.] [RT36-Z1]

## Discussion

Information that was derived from interviews with local trappers regarding the evolution of marten populations and pelt counts had exhibited the same trends as fur sales data that were compiled by the Government of Quebec (Ministry of Forests, Wildlife and Parks, MFFPQ) at the regional level (Fig. 2). Fisher abundance appears to have increased, especially since the 2000s at the northern limit of its range (Zones 2 and 4). Compared to Zone 3, Zone 2 is a region that contains more agricultural operations and more mixedwood stands than coniferous stands. However, trappers have noted a recent decline in fisher populations, which is particularly pronounced in Zone 3. The main cause of this decline is attributed by trappers to the decrease in large conifers that are used as resting and rearing sites. Marten populations are decreasing throughout the study area. However, trappers have acknowledged the discrepancy between sales of pelts that were recorded for each trapline and what was actually taken. One of the main reasons is the harvest on unstructured territories and exchanges between trappers to respect harvesting thresholds set by the government that would permit them to retain their rights to exploitation. Interviews were also used to identify changes in populations of other species for which the Quebec Ministry of Forests, Wildlife and Parks did not have data, such as unexploited (e.g., prey) species or those for which the fur sales data do not reflect these observed changes (lynx exploitation thresholds), or the particular nature of a species (e.g., wolves, which trappers are targeting for the challenge that its capture represents).

Trappers who were interviewed in this study were concerned about marten populations, a species that is particularly vulnerable to habitat alterations caused by logging operations. Concerns were expressed about the lack of protective cover, which has been incurred through the increase in hardwood species [64] and reductions in coarse woody debris (CWD) on the ground surface [65, 66]. The marten has long been perceived as being dependent upon mature and aging conifer stands [3, 67]. These stands generally provide greater quantities of CWD than do younger stands, forest plantations and intensively managed stands [68]. CWD provides subnival spaces that can be used by marten [69]. Subnival spaces are particularly important for prey accessibility and provide protection against predation, together with resting sites for thermoregulation [70–73]. Consequently, habitat use by marten is mainly dependent upon the internal structure of the forest [74, 75]. Fishers, in contrast, appear to be able to exploit a greater diversity of habitats, including more open forest environments, the edges of agricultural environments, and even urban areas [22, 76]. Fishers are not necessarily old-growth forest specialists, and are known to utilize young forests, mixedwood stands and ecotones [22]. A study that was conducted in 2016 in New York State [77], however, showed that occupation of the territory by the fisher was positively influenced by proportions of coniferous and mixedwood forests, but negatively affected by the density and proportion of agricultural environments. Like marten, the fisher likely would be more dependent upon the structure of the forest than on the age or type of settlement [22, 73–75]. Participants also pointed out that mustelid habitats were especially consistent with those of their main prey: snowshoe hares, squirrels and other small mammals. These prey species and, therefore, fisher and marten, require coarse woody debris, vegetation cover, and structurally complex forests [72, 78]. This complex structure is encountered mainly in old-growth forests, but may also be present in younger stands that have suffered windfalls or insect outbreaks [79, 80].

Participants stressed the importance of the availability of resting and rearing sites, which they did not consider to be sufficiently protected by forest management. Fishers depend upon the availability of large moribund trees that often have rotting heartwood, such as white pine, white cedar and yellow birch, in which they can make their dens and raise their young [81, 82]. As noted by Bridger et al. in 2016 [22], managers need to consider the importance of den availability for martens and fishers. The scarcity of dead wood is a further ecological challenge to forest management [80, 83–85]. Protection measures for dead wood were previously mainly guided by FSC (Forest Stewardship Council) forest certification [86]. With the introduction of ecosystem-based forest management, silvicultural practices have tended to create forest landscapes that contain all of the diversity of natural forests, including the composition and shape of stands and the presence of trees of different sizes, snags or woody debris [87–90]. Participants also encourage forest development that creates small openings, similar to gaps that naturally drive the development of sugar maple-yellow birch stands [43].

More generally, participants reported that the return of martens after logging was more rapid in mixedwood than in softwood stands. Most mixedwood harvest cuts are partial cuts, while softwood stands are cut more intensively. In 2005, Potvin et al. [91] determined that cut mixedwood stands had higher lateral cover and higher regeneration compared to cut conifer stands. Forest management influences populations of competitors and even predators of marten and fisher. Lynx, wolf and coyote probably benefited indirectly from forest management, which favoured their main prey, including snowshoe hare and moose [91–93]. Marten would thus be the victim of apparent competition [94]. Whether through habitat alterations or its effects on prey or predator species, forest management appears to be detrimental to marten, while favouring the fisher.

The fisher is limited in its movements over snowpacks that do not fully support its weight and is dependent upon the coniferous cover that intercepts the snow [95]. However, lower snow accumulation and ice crusting over a longer period of time result in greater load-bearing capacity of the snowpack, which seems to favour the fisher, thereby providing it with access to new habitats [8, 96]. The experience of trappers also has made it possible to document this snow load-bearing capacity, a characteristic for which no scientific follow-ups have been performed over long temporal and spatial scales.

Climate change and human activity have led to changes in abundances of other species, which in turn may influence the population dynamics of fisher and marten. Raccoon and coyote populations have increased in the northern part of their ranges [97, 98]. Populations of bald eagles and other raptor species have appeared to be recovering naturally throughout North America, since the use of DDT was discontinued [99, 100]. All of these changes appear to be detrimental to marten, which would increase the abundance of predators and competitors while leading to marten habitat degradation at the same time. The fisher would be less affected, having fewer predators than marten.

The fisher may exert competitive dominance, thereby excluding marten from areas that the latter occupies [101]. Indeed, competition between the two species has been reported by the participants. Yet interviews with trappers indicated that the principal relationship between fishers and martens was predation by the former on the latter species. To our knowledge, no study has yet been able to evaluate effectively the extent of predation of fisher on marten. In 2010, McCann et al. [102] documented predation by fishers on martens in northern Wisconsin, which would occur only during the winter. Both fisher and marten eat squirrels, other small mammals and snowshoe hares, but in different proportions. They explained that their overlapping diets may lead to greater interactions in the winter when hunting by both predators decreases prey populations [103, 104]. Additional studies would be required to identify the importance of marten in the diet of fisher in areas where the species are sympatric. Assessment of predation by fisher on marten would be all the more relevant as the abundance of fisher increases north of its current range, resulting in greater interactions with marten.

In this study, it was difficult to distinguish between effects of natural and anthropogenic disturbances on the population dynamics of marten and fisher. Whether it is forest rejuvenation, increasing the proportion of deciduous species, intensifying agricultural activities, climate change, or the consequences of their effects on other animal species, all of these factors are cumulative and, according to the experiences of the trappers who were interviewed, appear to favour the fisher to the detriment of the marten.

## Conclusion

Trappers‘experience has shown that martens and fishers are not exclusive to coniferous stands, although the marten is more closely associated with these forests than is the fisher. Trappers also identified the fisher as an important predator of martens. The fisher appears to benefit from open environments, including landscapes that have undergone extensive human alteration, such as forest cut-overs and agroforestry systems. For example, forest management frequently affects the marten, but has much less of an effect on the fisher. Climate change is likely to benefit the fisher by facilitating snow conditions that are favorable to its movement. Forest management and climate change therefore benefit the fisher, to the detriment of the marten, which is facing habitat declines, increases in predation, and competition from the fisher and other carnivores.

## Appendix

### Interview guide

On which trapline and for how long have you been trapping?

How do you trap marten?

How do you install your traps?

Where do you set your traps?

What bait do you use? Do you use lures?

How do you trap fisher?

What do you think about:

The preys of marten?

The predators of marten?

The preys of fisher?

The predators of fisher?

What other species do you trap?

Have you noted any changes in your marten catches? And those of fishers?

If yes, what could be the causes?

Has there been logging on your trapline?

What are the important elements to preserve for marten and fisher habitats?

How should we manage the forest?

What time of the year do you trap?

Have you noticed changes in winter conditions?

Have you noticed changes in fur quality?

Have you noticed any changes in populations of other wildlife species?

## References

[1] Buskirk SW (1992). Conserving circumpolar forests for martens and fishers. Conserv Biol.

[2] Proulx G: The impact of human activities on North America mustelids. In Mustelids in a modern world: management and conservation aspects of small carnivore: human interactions. Edited by Griffiths HI. Leiden, The Netherlands: Backhuys Publishers; 2000: 53–75.

[3] Cheveau M, Imbeau L, Drapeau D, Bélanger L (2013). Marten space use and habitat selection in managed coniferous boreal forests of eastern Canada. J Wildl Manag.

[4] Wiebe PA, Fryxell JM, Thompson ID, Borger L, Baker JA (2013). Do trappers understand marten habitat?. J Wildl Manag.

[5] Lodge DM: Species invasions and deletions: community effects and responses to climate and habitat change. In Biotic interactions and global change. Edited by Kareiva PM, Kingsolver JG, Huey RB. Sunderland, Massachusetts, USA: Sinauer Associates; 1993: 367–387.

[6] Manlick PJ, Woodford JE, Zuckerberg B, Pauli JN (2017). Niche compression intensifies competition between reintroduced American martens (Martes Americana) and fishers (Pekania pennanti). J Mammal.

[7] Graham RW, Grimm EC (1990). Effects of global climate change on the patterns of terrestrial biological communities. Trends in Ecology and Evolution.

[8] Wilsey CB, Lawler JJ, Freund JA, Hagmann K, Hutten KM, McKenzie D, Townsend PA, Gwozdz R (2013). Tools for assessing climate impacts on fish and wildlife. Journal of Fish and Wildlife Management.

[9] Schneider SH, Root T (2002). Wildlife responses to climate change: north American case studies.

[10] Johnson CJ: Regulating and planning for cumulative effects: the Canadian experience. In Cumulative effects in wildlife management: impact mitigation. Edited by Krausman PR, Harris LK. Boca Raton, Florida, USA: CRC Press; 2011: 29–46.

[11] Gallant D, Gauvin LY, Berteaux D, Lecomte N (2016). The importance of data mining for conservation science: a case study on the wolverine. Biodivers Conserv.

[12] Becker EF (1991). A terrestrial furbearer estimator based on probability sampling. J Wildl Manag.

[13] Hiller TL, Etter DR, Belant JL, Tyre AJ (2011). Factors affecting harvests of fishers and American martens in northern Michigan. J Wildl Manag.

[14] Gese EM: Monitoring of terrestrial carnivore populations. In Carnivore conservation. Edited by Gittleman JL, Funk SM, MacDonald DW, Wayne RK: Cambridge University Press & The Zoological Society of London; 2001: 372–396.

[15] Poulin J-F, Jolicoeur H, Canac-Marquis P, Larivière S: Investigation sur les facteurs à l’origine de la hausse de la récolte de pékans (Martes pennanti) au Québec depuis 1984. pp. 71: Ministère des Ressources naturelles et de la Faune d Québec, Direction du développement de la faune et Université du Québec à Rimouski, Département de biologie et des sciences de la santé; 2006:71.

[16] Kawaguchi T, Desrochers A, Bastien H (2015). Snow tracking and trapping harvest as reliable sources for inferring abundance: a 9-year comparison. Northeast Nat.

[17] Erickson DW: Estimating and using furbearer harvest information. In Midwest furbearer management. Edited by Sanderson GC. Wichita, Kansas, USA: Proceedings of the Symposium, 43rd Midwest Fish and Wildlife Conference; 1982: 53–66.

[18] Daigle JJ, Muth RM, Dwick RR, Glass RJ (1998). Sociocultural dimensions of trapping: a factor analysis study of trappers in six northeastern states. Wildl Soc Bull.

[19] Dorendorf RR (2016). Fix PJ.

[20] Strickland MA: Harvest management of fishers and American marten. In Martens, sables, and fishers: biology and conservation. Edited by Buskirk SW, Harestad AS, Raphael MG, Powell RA. Ithaca & London: Cornell University Press; 1994: 149–164.

[21] Berkes F, Berkes MK (2009). Ecological complexity, fuzzy logic, and holism in indigenous knowledge. Futures.

[22] Bridger MC, Johnson CJ, Gillingham MP (2016). Assessing cumulative impacts of forest development on the distribution of furbearers using expert-based habitat modeling. Ecol Appl.

[23] Cheveau M, Imbeau L, Drapeau D, Bélanger L (2008). Current status and future directions of traditional ecological knowledge in forest management: a review. For Chron.

[24] Asselin H: Indigenous forest knowledge. In Routledge Handbook of Forest Ecology. Edited by Peh K, Corlett R, Bergeron Y. New York: Routledge; 2015: 586–596s.

[25] Tendeng B, Asselin H, Imbeau L (2016). Moose (Alces Americanus) habitat suitability in temperate deciduous forests based on Algonquin traditional knowledge and on a habitat suitability index. Ecoscience.

[26] Watson A, Alessa L, Glaspell B. The relationship between traditional ecological knowledge, evolving cultures, and wilderness protection in the circumpolar north. Conserv Ecol. 2003;8(1):2. [online] URL: http://www.consecol.org/vol8/iss1/art2/.

[27] Stevenson M (2005). Traditional knowledge and sustainable forest management.

[28] Drapeau P, Leduc A, Bergeron Y: Bridging ecosystem and multiple species approaches for setting conservation targets in managed boreal landscapes. In Setting conservation targets in managed forest landscapes. Edited by Villard M-A, Jonsson BG. New-York, USA: Cambrige University Press; 2009: 129–160.

[29] McLaren MA, Thompson ID, Baker JA (1998). Selection of vertebrate wildlife indicators for monitoring sustainable forest management in Ontario. For Chron.

[30] Cheveau M: z6.

[31] Obbard ME, Jones JG, Newman R, Booth A, Satterthwaite AJ, Linscombe G: Furbearer harvests in North America. In Wild furbearer management and conservation in North America. Edited by Novak M, Baker JA, Obbard ME, Malloch B. Toronto, Canada: Ontario Ministry of Natural Resources; 1987: 1007–1034.

[32] ÉcoRessources: L’industrie faunique comme moteur économique régional. Une étude ventilant par espèce et par région les retombées économiques engendrées par les chasseurs, les pêcheurs et les piégeurs québécois en 2012. Rapport technique présenté au Ministère des Forêts, de la Faune et des Parcs du Québec; 2014.

[33] Caduto MJ, Bruchac J. How fisher went to the skyland: the origin of the big dipper. In Keepers of the Earth: ative american stories and environmental activities for children. 1988:117–25.

[34] Cheveau M. Effets multiscalaires de la fragmentation de la forêt par l’aménagement forestier sur la martre d’Amérique en forêt boréale de l’est du Canada: Ph. D*.* Université du Québec en Abitibi-Témiscamingue; 2010.

[35] Garibaldi A, Turner N (2004). Cultural keystone species: implications for ecological conservation and restoration. Ecol Soc.

[36] Powell R (1993). The fisher: life history, ecology and behavior.

[37] Zielinski WJ, Tucker JM, Rennie KM (2017). Niche overlap of competing carnivores across climatic gradients and the conservation implications of climate change at geographic range margins. Biol Conserv.

[38] Sweitzer RA, Furnas BJ, Barrett RH, Purcell KL, Thompson CM (2016). Landscape fuel reduction, forest fire, and biophysical linkages to local habitat use and local persistence of fishers (Pekania pennanti) in sierra Nevada mixed-conifer forests. For Ecol Manag.

[39] Powell RA, Buskirk SW, Zielinski WJ: Fisher and marten (*Martes pennanti and Martes americana*). In Wild mammals of North America*.* Edited by Feldhamer G, Thompson B, Chapman J. Baltimore, MD: Johns Hopkins University Press; 2003: 635–649.

[40] Lewis JC, A., Powell R, Zielinski WJ (2012). Carnivore translocations and conservation: insights from population models and field data for fishers (*Martes pennanti***)**. PLoS One.

[41] Logan T, Charron I, Chaumont D, Houle D: Atlas of climate scenarios for Québec forests. pp. 57 + annexes: Ouranos et MRNF; 2011:57 + annexes.

[42] Saucier J-P, Bergeron J-F, Grondin P, Robitaille A (1998). **Les régions écologiques du Québec méridional (3e version): un des éléments du système hiérarchique de classification écologique du territoire mis au point par le ministère des Ressources naturelles du Québec**. L’aubelle.

[43] Després T, Asselin H, Doyon F, Drobyshev I, Bergeron Y (2017). Gap dynamics of late successional sugar maple-yellow birch forests at their northern range limit. J Veg Sci.

[44] MFFP: Système d’information sur les animaux à fourrure. Base de données des ventes de fourrures au Québec.: Ministère des Forêts, de la Faune et des Parcs; 2017.

[45] Grenier D, Bergeron Y, Kneeshaw D, Gauthier S (2005). Fire frequency for the transitional mixedwood forest of Timiskaming, Quebec, Canada. Can J For Res.

[46] Bouchard M, Kneeshaw D, Bergeron Y (2006). Forest dynamics after successive spruce budworm outbreaks in mixedwood forests. Ecology.

[47] Morin H (1994). Dynamics of balsam fir forests in relation to spruce budworm outbreaks in the boreal zone of Quebec. Can J For Res.

[48] Bergeron Y, Flannigan M, Gauthier S, Leduc A, Lefort P (2004). Past, current and future fire frequency in the Canadian boreal forest: implications for sustainable forest management. AMBIO: A Journal of the Human Environment.

[49] Morin H, Laprise D, Simard A-A, Amouch S: Régime des épidémies de la tordeuse des bourgeons de l’épinette dans l’Est de l’Amérique du Nord. In Aménagement écosystémique en forêt boréal*e.* Edited by Gauthier S, Vaillancourt M-A, Leduc A, Grandpré LD, Kneeshaw D, Morin H, Drapeau P, Bergeron Y. Québec, Qc: Presses de l’Université du Québec; 2008: 165–192.

[50] Ndione PD: Les impacts de la foresterie industrielle sur les activités traditionnelles autochtones en forêt tempérée mixte. Ph. D. Université du Québec en Abitibi-Témiscamingue,, Sciences en environnement; 2014.

[51] IFC: Statistiques de production de fourrures sauvages 2010–2011 à 2014-2015**.** Institut de la fourrure du Canada; 2016.

[52] Évolution des ventes de permis de chasse, de pêche et de piégeage – Vente de permis de piégeage par catégories. [http://mffp.gouv.qc.ca/faune/statistiques/vente-permis-piegeage.jsp].

[53] Papatie J: Vécu et réflexion de la communauté Anicinapek de Kitcisakik avec le régime forestier des Québécois**.** pp. 27: Conseil des Anicinapek de Kitcisakik. Déposé par les Anicinapek de Kitcisakik, James Papatie, Ogima, dans le cadre de la Commission d'étude sur la gestion de la forêt publique québécoise.; 2004:27.

[54] Saint-Arnaud M, Asselin H, Dubé C, Croteau Y, Papatie C: Developing criteria and indicators for aboriginal forestry : mutual learning through collaborative research. In Changing the culture of forestry in Canada: building effective institutions for aboriginal engagement in sustainable forest management. Edited by Stevenson MG, Natcher DC. Edmonton: CCI Press and Sustainable Forest Management Network; 2009: 85–105.

[55] Joshi L, Arévalo L, Luque N, Alegre J, Sinclair F: Local ecological knowledge in natural resource management. In Proceedings, Bridging scales and epistemologies: Linking local knowledge and global science in multi-scale assessments. Alexandria, Egypt; 2004.

[56] IFC: Sustainable forest management for timber, furbearer and forest biodiversity: a guide for trappers, furbearer and forest managers. pp. 36: Institut de la fourrure du Canada; 2006:36.

[57] Davis A, Wagner JR (2003). Who knows? On the importance of identifying “experts” when researching local ecological knowledge. Hum Ecol.

[58] Gamborg C, Parsons R, Puri RK, Sandøe P: Ethics and research methodologies for the study of traditional forest-related knowledge. In Traditional forest-related knowledge: sustaining communities, ecosystems and biocultural diversity. Edited by Parrotta JA, Trosper RL: World Forest XII, IUFRO, The Christensen Fund & Springer; 2012: 535–562.

[59] Goodman LA (1961). Snowball sampling. Ann Math Stat.

[60] Patton MQ (2002). Qualitative research and evaluation methods.

[61] Rousseau MH (2008). L’acceptabilité sociale de l’aménagement forestier sur l’île d’Anticosti, un territoire à vocation faunique.

[62] Asselin H, Basile S (2012). Éthique de la recherche avec les Peuples autochtones: qu'en pensent les principaux intéressés?. Éthique publique.

[63] Deschenaux F: Guide d'introduction au logiciel QSR Nvivo 7.: Association pour la recherche qualitative. Trois-Rivières, Québec.; 2007.

[64] Danneyrolles V, Arseneault D, Bergeron Y (2016). Long-term compositional changes following partial disturbance revealed by the resurvey of logging concession limits in the northern temperate forest of eastern Canada. Can J For Res.

[65] Bowman JC, Sleep D, Forbes GJ, Edwards M (2000). The association of small mammals with coarse woody debris at log and stand scales. For Ecol Manag.

[66] Fauteux D, Mazerolle MJ, Imbeau L, Drapeau P (2013). Site occupancy and spatial co-occurrence of boreal small mammals are favoured by late-decay woody debris. Can J For Res.

[67] Strickland MA, Douglas CW: Martes. In Wild furbearer management and conservation in north America. Edited by Novak M. Ottawa, Ontario, Canada: Ministry of Natural Resources; 1987: 530–546.

[68] Hély C, Bergeron Y, Flannigan MD (2000). Coarse woody debris in the southeastern Canadian boreal forest: composition and load variations in relation to stand replacement. Can J For Res.

[69] Corn JG, Raphael MG (1992). Habitat characteristics at marten subnivean access sites. J Wildl Manag.

[70] Buskirk SW, Harlow HJ, Forest SC (1988). Temperature regulation in American marten (Martes americana) in winter. Natl Geogr Res.

[71] Sherburne SS, Bissonette JA (1994). Marten subnivean access point use: response to subnivean prey levels. J Wildl Manag.

[72] Chapin TG, Hanison DJ, Phillips DM (1997). Seasonal habitat selection by marten in an untrapped forest preserve. J Wildl Manag.

[73] Vigeant-Langlois C, Desrochers A (2011). Movements of wintering American marten (*Martes americana*): relative influences of prey activity and forest stand age. Can J For Res.

[74] Coffin KW, Kujala QJ, Douglass RJ, Irby LR: Interactions among marten prey availability, vulnerability, and habitat structure. In Martes: Taxonomy, ecology, techniques, and management*.* Edited by Proulx G, Bryant HN, Woodard PM. Edmonton (Alberta, Canada): The Provincial Museum of Alberta; 1997: 199–210.

[75] Payer DC, Harrison DJ (2003). Influence of forest structure on habitat use by American marten in an industrial forest. For Ecol Manag.

[76] LaPoint S, Gallery P, Wikelski M, Kays R (2013). Animal behavior, cost-based corridor models, and real corridors. Landsc Ecol.

[77] Fuller AK, Linden DW, Royle JA (2016). Management decision making for fisher populations informed by occupancy modeling. J Wildl Manag.

[78] Weir RD, Harestad AS (2003). Scale-dependent habitat selectivity by fishers in south-central British Columbia. J Wildl Manag.

[79] Potvin F, Bélanger L, Lowell K (2000). Marten habitat selection in a clearcut boreal landscape. Conserv Biol.

[80] Siitonen J, Martikainen P, Punttila P, Rauh J (2000). Coarse woody debris and stand characteristics in mature managed and old-growth boreal mesic forests in southern Finland. For Ecol Manag.

[81] Weir RD, Phinney M, Lofroth EC (2012). Big, sick, and rotting: why tree size, damage, and decay are important to fisher reproductive habitat. For Ecol Manag.

[82] Uprety Y, Asselin H, Bergeron Y (2013). Cultural importance of white pine (*Pinus strobus L.*) to the Kitcisakik Algonquin community of western Quebec, Canada. Can J For Res.

[83] McCarthy BC, Bailey RR (1994). Distribution and abundance of coarse woody debris in a managed forest landscape of the central Appalachians. Can J For Res.

[84] Fridman J, Walheim M (2000). Amount, structure, and dynamics of dead wood on managed forestland in Sweden. For Ecol Manag.

[85] Angers V-A: L’enjeu écologique du bois mort – Complément au Guide pour la description des principaux enjeux écologiques dans les plans régionaux de développement intégré des ressources et du territoire. pp. 45: Ministère des Ressources naturelles et de la Faune du Québec, Direction de l’environnement et de la protection des forêts, Gouvrenement du Québec; 2009:45.

[86] FSC: National Boreal Standard. pp. 181: Forest Stewardship Council Canada; 2004:181.

[87] Beese WJ, Dunsworth BG, Zielke K, Bancroft B (2003). Maintaining attributes of old-growth forests in coastal B.C. through variable retention. For Chron.

[88] Gauthier S, Vaillancourt M-A, Leduc A, Grandpré LD, Kneeshaw D, Morin H, Drapeau P, Bergeron Y (2008). Aménagement écosystémique en forêt boréale.

[89] Kuuluvainen T (2009). Forest management and biodiversity conservation based on natural ecosystem dynamics in northern Europe: the complexity challenge. AMBIO: A Journal of the Human Environment.

[90] MFFP: Stratégie d'aménagement durable des forêts**.** (Ministère des Forêts de la Faune et des Parcs du Québec ed.: Bibliothèque et Archives nationales du Québec; 2015.

[91] Potvin F, Breton L, Courtois R (2005). Response of beaver, moose, and snowshoe hare to clear-cutting in a Quebec boreal forest: a reassessment 10 years after cut. Can J For Res.

[92] Lesmerises F, Dussault C, St-Laurent M-H (2012). Wolf habitat selection is shaped by human activities in a highly managed boreal forest. For Ecol Manag.

[93] Simard V (2016). Impact à moyen et long terme des coupes de jardinage sur l’habitat d’hiver du lièvre d’Amérique (*Lepus americanus*) en érablière à bouleau jaune.

[94] Holt RD (1984). Spatial heterogeneity, indirect interactions, and the coexistence of prey species. Am Nat.

[95] Raine MA (1987). Winter food habits and foraging behaviour of fishers (*Martes pennanti*) and martens (*Martes americana*) in southeastern Manitoba. Can J Zool.

[96] Kilpatrick HJ, Rego PW (1994). Influence of season, sex, and site availability on fisher (*Martes pennanti*) rest-site selection in the central hardwood forest. Can J Zool.

[97] Randa LA, Yunger JA (2006). Carnivore occurrence along an urban-rural gradient: a landscape-level analysis. J Mammal.

[98] Boisjoly D, Ouellet J-P, Courtois R (2010). Coyote habitat selection and management implications for the Gaspésie caribou. J Wildl Manag.

[99] Grier JW (1982). Ban of DDT and subsequent recovery of reproduction in bald eagles. Science.

[100] Kirk DA, Hyslop C (1998). Population status and recent trends in Canadian raptors: a review. Biol Conserv.

[101] Fisher JT, Anholt B, Bradbury S, Wheatley M, Volpe JP (2013). Spatial segregation of sympatric marten and fishers: the influence of landscapes and species-scapes. Ecography.

[102] McCann NP, Zollner PA, Gilbert JH (2010). Survival of adult martens in northern Wisconsin. J Wildl Manag.

[103] Giuliano WM, Litvaitis JA, Stevens CL (1989). Prey selection in relation to sexual dimorphism of fishers (*Martes pennanti*) in New Hampshire. J Mammal.

[104] Cumberland RE, Dempsey JA, Forbes GJ (2001). Should diet be based on biomass? Importance of larger prey to the American marten. Wildl Soc Bull.

